# Supplementary material for: From sequence to enzyme mechanism using multi-label machine learning
Source: BMC Bioinformatics. 2014 May 19;15:150. doi: 10.1186/1471-2105-15-150 (PMC4229970; doi:10.1186/1471-2105-15-150)
Supplement: Additional file 2 — Java code of ml2db. Additional file ml2db_code.tar.gz contains the Java source code to run the multi-label machine learning experiments and save the results to database. The code’s Javadoc is included. [file 1471-2105-15-150-S2.zip › additional file 2/ml2db/ml2db/doc/main/ml2db/mulan/learn/evaluation/EvaluatorNoAUC.html]

EvaluatorNoAUC


JavaScript is disabled on your browser.


- Overview
- Package
- Class
- Use
- Tree
- Deprecated
- Index
- Help

- Prev Class
- Next Class

- Frames
- No Frames

- All Classes

- Summary:
- Nested |
- Field |
- Constr |
- Method

- Detail:
- Field |
- Constr |
- Method


main.ml2db.mulan.learn.evaluation

## Class EvaluatorNoAUC

- java.lang.Object
- - mulan.evaluation.Evaluator
  - - main.ml2db.mulan.learn.evaluation.EvaluatorNoAUC

- Direct Known Subclasses:
  :   CrossEvaluator

  ---

    

  ```
  public abstract class EvaluatorNoAUC
  extends mulan.evaluation.Evaluator
  ```

  Prepares the evaluation measures taking into consideration the dataset size.
  MicroAUC and MacroAUC are \*not\* calculated for datasets > n instances.
  Predictions are emitted and saved to database from within the evaluation
  procedure (predictions have to be generated for calculating the evaluation
  metrics anyway...)

  Version:
  :   7 Apr 2011

  Author:
  :   Luna De Ferrari ldf(at)st-andrews.ac.uk

- - ### Constructor Summary

    Constructors

    Constructor and Description

    `EvaluatorNoAUC(CrossExperimenter experimenter,
    boolean strict)`
  - ### Method Summary

    Methods

    | Modifier and Type | Method and Description |
    | --- | --- |
    | `mulan.evaluation.Evaluation` | `evaluate(mulan.classifier.MultiLabelLearner learner, mulan.data.MultiLabelInstances data, boolean savePredictions, PredictionsSaver predSaver)` Evaluates a `MultiLabelLearner` on given test data set. |
    | `mulan.evaluation.Evaluation` | `evaluate(mulan.classifier.MultiLabelLearner learner, mulan.data.MultiLabelInstances data, java.util.List<mulan.evaluation.measure.Measure> measures, boolean savePredictions, PredictionsSaver predSaver)` Evaluates a `MultiLabelLearner` on given test data set using specified evaluation measures |
    | `java.lang.String` | `getEndTime()` |
    | `CrossExperimenter` | `getExperimenter()` |
    | `java.lang.String` | `getStartTime()` |
    | `static boolean` | `getStrictness()` |
    | `boolean` | `isStrict()` |
    | `void` | `setEndTime(java.lang.String endTime)` |

    - ### Methods inherited from class mulan.evaluation.Evaluator

      `checkData, checkLearner, crossValidate, crossValidate, evaluate, evaluate, setSeed`
    - ### Methods inherited from class java.lang.Object

      `equals, getClass, hashCode, notify, notifyAll, toString, wait, wait, wait`

- - ### Constructor Detail


    - #### EvaluatorNoAUC

      ```
      public EvaluatorNoAUC(CrossExperimenter experimenter,
                    boolean strict)
      ```
  - ### Method Detail


    - #### evaluate

      ```
      public mulan.evaluation.Evaluation evaluate(mulan.classifier.MultiLabelLearner learner,
                                         mulan.data.MultiLabelInstances data,
                                         boolean savePredictions,
                                         PredictionsSaver predSaver)
                                           throws java.lang.IllegalArgumentException,
                                                  java.lang.Exception
      ```

      Evaluates a `MultiLabelLearner` on given test data set.

      Parameters:
      :   `learner` - the learner to be evaluated
      :   `data` - the data set for evaluation
      :   `predSaver` -

      Returns:
      :   the evaluation result

      Throws:
      :   `java.lang.IllegalArgumentException` - if either of input parameters is null.
      :   `java.lang.Exception`


    - #### evaluate

      ```
      public mulan.evaluation.Evaluation evaluate(mulan.classifier.MultiLabelLearner learner,
                                         mulan.data.MultiLabelInstances data,
                                         java.util.List<mulan.evaluation.measure.Measure> measures,
                                         boolean savePredictions,
                                         PredictionsSaver predSaver)
                                           throws java.lang.IllegalArgumentException,
                                                  java.lang.Exception
      ```

      Evaluates a `MultiLabelLearner` on given test data set using
      specified evaluation measures

      Parameters:
      :   `learner` - the learner to be evaluated via cross-validation
      :   `data` - the data set for cross-validation
      :   `measures` - the evaluation measures to compute

      Returns:
      :   an Evaluation object

      Throws:
      :   `java.lang.IllegalArgumentException` - if an input parameter is null
      :   `java.lang.Exception`


    - #### getEndTime

      ```
      public java.lang.String getEndTime()
      ```


    - #### getExperimenter

      ```
      public CrossExperimenter getExperimenter()
      ```


    - #### getStartTime

      ```
      public java.lang.String getStartTime()
      ```


    - #### isStrict

      ```
      public boolean isStrict()
      ```


    - #### setEndTime

      ```
      public void setEndTime(java.lang.String endTime)
      ```


    - #### getStrictness

      ```
      public static boolean getStrictness()
      ```


- Overview
- Package
- Class
- Use
- Tree
- Deprecated
- Index
- Help

- Prev Class
- Next Class

- Frames
- No Frames

- All Classes

- Summary:
- Nested |
- Field |
- Constr |
- Method

- Detail:
- Field |
- Constr |
- Method
